# Antidepressant and Neurocognitive Effects of Isoflurane Anesthesia versus Electroconvulsive Therapy in Refractory Depression

**DOI:** 10.1371/journal.pone.0069809

**Published:** 2013-07-26

**Authors:** Howard R. Weeks, Scott C. Tadler, Kelly W. Smith, Eli Iacob, Mikala Saccoman, Andrea T. White, Joshua D. Landvatter, Gordon J. Chelune, Yana Suchy, Elaine Clark, Michael K. Cahalan, Lowry Bushnell, Derek Sakata, Alan R. Light, Kathleen C. Light

**Affiliations:** 1 Department of Psychiatry, University Neuropsychiatric Institute, University of Utah School of Medicine, Salt Lake City, Utah, United States of America; 2 Department of Anesthesiology, University of Utah School of Medicine, Salt Lake City, Utah, United States of America; 3 Neuroscience Program, University of Utah School of Medicine, Salt Lake City, Utah, United States of America; 4 Department of Educational Psychology, College of Education, University of Utah, Salt Lake City, Utah, United States of America; 5 Department of Exercise and Sport Science, College of Health, University of Utah, Salt Lake City, Utah, United States of America; 6 Department of Neurology and Center for Alzheimer's Care, Imaging and Research, University of Utah School of Medicine, Salt Lake City, Utah, United States of America; 7 Department of Psychology, College of Social and Behavioral Science, University of Utah, Salt Lake City, Utah, United States of America; Peking University, China

## Abstract

**Background:**

Many patients have serious depression that is nonresponsive to medications, but refuse electroconvulsive therapy (ECT). Early research suggested that isoflurane anesthesia may be an effective alternative to ECT. Subsequent studies altered drug, dose or number of treatments, and failed to replicate this success, halting research on isoflurane's antidepressant effects for a decade. Our aim was to re-examine whether isoflurane has antidepressant effects comparable to ECT, with less adverse effects on cognition.

**Method:**

Patients with medication-refractory depression received an average of 10 treatments of bifrontal ECT (n = 20) or isoflurane (n = 8) over 3 weeks. Depression severity (Hamilton Rating Scale for Depression-24) and neurocognitive responses (anterograde and retrograde memory, processing speed and verbal fluency) were assessed at Pretreatment, Post all treatments and 4-week Follow-up.

**Results:**

Both treatments produced significant reductions in depression scores at Post-treatment and 4-week Follow-up; however, ECT had modestly better antidepressant effect at follow-up in severity-matched patients. Immediately Post-treatment, ECT (but not isoflurane) patients showed declines in memory, fluency, and processing speed. At Follow-up, only autobiographical memory remained below Pretreatment level for ECT patients, but isoflurane patients had greater test-retest neurocognitive score improvement.

**Conclusions:**

Our data reconfirm that isoflurane has an antidepressant effect approaching ECT with less adverse neurocognitive effects, and reinforce the need for a larger clinical trial.

## Introduction

Although antidepressant medications are effective for many patients with depression, the rate of response to the first agent administered can be as low as 50%. For nonresponders to the first agent, the Sequenced Treatment Alternatives to Relieve Depression (STAR*D) trial showed that the remission rate decreased from 37% to 13% as successive medication alternatives were tried [1] . Electroconvulsive therapy (ECT) is generally acknowledged to be the most effective treatment for severe and medication-refractory depression [2]. Its remission rates are between 55–90%, even in patients whose depression fails to remit with multiple trials of medications [3], [4], [5]. Significant reduction in symptoms also occurs more rapidly with ECT than medications, typically in 2–4 weeks consisting of 8–14 treatment sessions. Though effective, ECT is associated with significant adverse cognitive effects such as retrograde amnesia, problems with concentration and attention, and other cognitive sequelae. With the notable exception of autobiographical memory problems, most adverse cognitive effects of ECT are reported to resolve within 2 weeks [6]. Bifrontal ECT is equally effective to the more traditional bitemporal ECT and has less memory disruption [7]. There is also a widespread public misunderstanding that the seizure induced by ECT may be painful and traumatic, and carries high risk of brain damage and personality change, making patients and family members reluctant to approve this treatment option [8]. For these reasons, this effective therapy has become a treatment of last resort (relegated to 5th, 6th or 7th step after the failure of other therapies per American Psychiatric Association guidelines) and ECT is administered to only about 100,000 patients a year [8]. Thus, millions of patients suffering from major depression do not receive effective therapy, and others receive relief only after many months of medication trials.

It would be highly desirable to establish an alternative therapy that has similar benefits to ECT while minimizing any adverse neurocognitive effects and having wider social acceptability. One alternative meeting these criteria that has some prior data supporting its potential effectiveness in depression is deep inhalation anesthesia with isoflurane (ISO). Like ECT, deep anesthesia with ISO induces a brief state of electrocortical quiescence (burst suppression on electroencephalogram (EEG)), but does so without inducing convulsions or other seizure symptoms. Based on two preliminary studies directed by Langer, et al in Europe [9],[10], a series of 6 treatments with ISO had similar efficacy to 6 ECT sessions in reducing depressive symptoms without causing memory loss. The first study was a pilot study showing reduction of depressive symptoms in 11 patients exposed to variable numbers of ISO treatments, and the second was a small double-blind study comparing 10 depressed women treated with ISO vs. 10 treated with ECT for 6 sessions. Assessments indicated cognitive improvement in the ISO group vs. declines in ECT group after treatment. Subsequent work by Engelhardt [11] demonstrated similar antidepressant effect with a combined series of ISO treatments followed by ECT. A small open-label report by Carl, et al. [12] also suggested similar antidepressant efficacy of deep ISO anesthesia compared to ECT. In contrast, however, Greenberg, et al. [13] showed relatively little improvement with ISO treatments in a pilot study involving 6 elderly depressed patients (including 5 aged 74–82 years). In another pilot study, Garcia-Toro, et al. [14] treated 10 patients with 4 treatments of low dose sevoflurane (approximately 2.5%). While a 24% reduction in depressive symptoms was noted on average, the clinical effect was not deemed sufficient after 4 treatments and patients were switched to ECT. In these negative studies, methodological concerns were that the number of treatments were fewer than are typically effective for ECT, treatment effects that initially seemed promising nevertheless were stopped, and in the latter study, a low concentration of sevoflurane was used instead of a high concentration of isoflurane. There was essentially a cessation of interest in this potential treatment until recently, when studies in animal models also indicated antidepressant effects of ISO anesthesia. In a pilot study in our lab, mice treated with a single dose of either high dose ISO or desipramine and assessed 24 hours later using the forced swim test showed reduced immobility times as compared to the control group, suggesting antidepressant efficacy [15]. To assess more enduring antidepressant effects, Wang, et al [16] administered isoflurane (2%) or halothane (1.5%) to adult male Sprague Dawley rats continuously for two hours. Two weeks later, in a learned helplessness paradigm, isoflurane-treated rats had fewer failure trials and faster mean escape latency than naïve controls in the shuttle box avoidance task. Halothane-treated rats showed no antidepressant effects, suggesting that the reduced expression of learned helplessness is specific to isoflurane rather than a general effect associated with exposure to volatile anesthetics.

Given the large number of patients with medication-refractory depression (including many who are under age 70, relatively healthy, and would be deemed good candidates for ISO anesthesia), and improvements in medical monitoring and management of patients during deep anesthesia, a renewed effort to evaluate ISO anesthesia as an effective alternative therapy to ECT is warranted. As a small open-label comparability study, the present study's primary objective was to examine whether deep ISO anesthesia shows similar efficacy to ECT in alleviating moderate to severe depression in medication-refractory patients. The secondary objective was to evaluate whether a series of 10 treatments with deep ISO anesthesia over a 3 week period results better neurocognitive function at 24–48 hours after the last treatment and 4 weeks later than treatments with bifrontal ECT, a form of ECT previously shown to have lesser effects on memory on cognition than bitemporal ECT [7].

## Materials and Methods

### Participants

Participants included 28 patients aged 18–65 years with moderate to severe depression (Hamilton Rating Scale for Depression HRSD-24 score of 20 or higher). They were recruited from among patients referred for ECT to the University of Utah Neuropsychiatric Institute (UNI) based on unsatisfactory response and/or intolerance to multiple antidepressant pharmacological interventions, and included 21 with a primary diagnosis of major depressive disorder (MDD) and 7 with a diagnosis of bipolar disorder (BPD). After evaluation by the study psychiatrist (HW), patients were offered enrollment and the opportunity to choose either the standard treatment, ECT, or the investigative treatment, ISO anesthesia. Exclusions included: 1) primary psychotic disorder, dysthymia, or personality disorder; 2) significant premorbid cognitive impairment (defined as Mini-mental Exam score below 24); 3) unstable cardiovascular or cerebrovascular disease, 4) any other contraindication to ISO anesthesia; 5) pregnancy or 6) inability to consent. Enrolled patients continued their usual pharmacotherapy and psychotherapy, except for anticonvulsants which were discontinued (see Table 1) while undergoing the study. In this open-label study, 20 patients were treated using the medical standard intervention, ECT, and 8 patients with the nonstandard intervention, ISO. Because initial tests revealed that the ECT patients had significantly greater depression severity than the ISO patients at Pretreatment, the responses of the ISO group were also compared to a severity-matched ECT subgroup (n = 8); this ECT-Matched subgroup also had the same ratio of patients with BPD vs. MDD diagnoses as the ISO group (3 BPD/5MDD). All patients completed both Pretreatment and Post-treatment testing. One patient in each group failed to return for 4-week Follow-up neurocognitive testing.

**Table 1:** Group Demographics, Intellectual Level, Depression Severity, and Pretreatment Medications.

	ECT-All	ECT-Matched	ISO
**Gender (Male/Total)**	12/20	6/8	3/8
**Diagnosis (MDD/BPD)**	16/4	5/3	5/3
**Mean Age (SEM)** a	41.60 (2.80)	41.38 (5.39)	35.38 (3.50)
**Mean WTAR (SEM)** a	107.90 (2.19)	107.88 (4.67)	117.00 (5.01)
**Depression Severity:**			
Pretreatment HRSD-24*	36.55 (2.08)	28.00 (2.20)	26.63 (1.42)
Post-Treatment HRSD-24^a^	10.05 (1.98)	9.25 (2.32)	12.50 (3.63)
Follow-up HRSD-24**	14.32 (2.14)	8.88 (2.27)	17.57 (2.82)
Post-Treatment % remission^a^	50%	50%	50%
Post-Treatment % responders^a^ *^,^* b	90%	88%	75%
**Pretreatment Medications (# of patients):**			
SSRI	9	5	2
SNRI	8	1	3
NRI	3	1	0
Lithium/valproate	5	1	2
Antipsychotic	8	2	5
Anticonvulsant^c^	14	6	2
No psychotropic meds	3	1	1

* *ECT-All vs. ISO, p<0.05; ECT-Matched vs. ISO nonsignificant.*

** *ECT-Matched vs. ISO, p<0.05; ECT-All vs. ISO non-significant.*

a *Group differences non-significant.*

b *Responders are defined as at least 50% reduction in HRSD score.*

c *Anticonvulsant medications were stopped during treatment sessions in all groups.*

### Ethics Statement

This study was approved by the University of Utah Institutional Review Board (IRB Protocol No. 00025750) and all participants provided written informed consent.

### Study Design

The design was an open-label, two-arm treatment trial, comparing 8–12 sessions of ECT vs. 10 sessions of ISO anesthesia treatments over a 2.5–3.5 week period. Primary outcome measures included: 1) depressive symptoms assessed by clinical assessment using the HRSD-24 by blinded investigators, and patient self-ratings on the 16-item Quick Inventory of Depressive Symptomatology Self Report (QIDS-SR16); 2) neurocognitive function assessed by tests of memory, executive function, and processing speed. HRSD-24 and cognitive assessments were completed 3 times: 1) Pretreatment, 2) 24–48 hours after the last treatment session (Post-treatment), and 3) 4 weeks after the last treatment session (Follow-up). In 12 ECT and all 8 ISO patients, QIDS-SR16 assessments of depression were obtained just prior to each treatment session, to provide a more continuous picture of the rate of symptom change.

### Treatment Procedures

Patients received standard monitoring as recommended by the American Society of Anesthesiologists (ASA). Procedures for the ECT treatment group: Induction was performed by an anesthesiologist (KS) with methohexital (1–3 mg/kg IV). A tourniquet was inflated around a hand followed by neuromuscular blockade using succinylcholine (adjusted by height, 1–2 mg/inch). Then, 60–120 seconds later, the bifrontal ECT stimulus was administered (SpECTrum 5000Q Electroconvulsive Therapy Unit, Mecta Corporation, Tualatin, OR). Visual evidence of tonic-clonic seizure was confirmed by observation in the hand that remained isolated from the systemic circulation. ECT procedure time averaged 20 min, with 30–45 min required for post-procedure recovery. ECT treatments were repeated approximately every other day for a total of 8–12 treatments (mean 9.6, standard error (SEM) 0.34), based on clinically determined need. Procedures for the ISO treatment group: As with ECT, anesthetic induction was achieved with methohexital (1–3 mg/kg IV) and neuromuscular blockade was accomplished with succinylcholine (adjusted by height, 1–2 mg/inch). Patients were infused with 500 ml of lactated Ringers solution to help reduce risk of hypotension. The patient's airway was secured via endotracheal intubation. The EEG-derived bispectral index (BIS) was monitored continuously using an Aspect A1000 monitor (BIS v. 3.3, Aspect Medical Systems, Newton MA), along with end-tidal concentration of isoflurane, carbon dioxide and oxygen. Isoflurane concentration was initially at 4% with high flow oxygen until >80% EEG burst suppression; it was then decreased to 2 times the age-adjusted Minimum Alveolar Concentration (MAC) for the patient and oxygen flows reduced. This level of burst suppression was maintained for 15 minutes (as per Langer, et al. [9], [10]) and then isoflurane was discontinued and the patient allowed to awaken. ISO procedure time averaged 40–45 min. Emergence from anesthesia was facilitated by the ANEclear**™** device (Anecare**_®_**, Salt Lake City, UT) that rapidly removes residual inhaled anesthestics, reducing post-procedure recovery time to 15–20 min. Significant hemodynamic alteration (change in heart rate or blood pressure more than 20% from baseline) was treated in standard fashion with appropriate agents (phenylephrine, intravenous fluids, etc.) as per the anesthesiologist's (KS's) discretion. ISO treatments were repeated approximately every other day for a total of 10 treatments.

### Neurocognitive Testing

At Pretreatment, all patients completed the Mini-mental Exam and the Wechsler Test of Adult Reading (WTAR) to confirm that intellectual levels were within normal limits and generally comparable across groups. Mini-mental scores were all >26 and did not differ for the ECT-All, ECT-Matched and ISO groups (means = 28.89, 28.94 and 29.22, respectively, p>0.38). WTAR scores and age also did not differ significantly (p>0.12; Table 1), but there was a weak trend toward higher WTAR and lower age in the ISO group. To ensure that our findings were not influenced by these subtle differences, both WTAR and age were retained as covariates in our group comparisons.

At Pretreatment, Post-treatment and Follow-up, the following battery of tests were completed. To assess retrograde amnesia, subjects completed the Autobiographical Memory Interview-Short Form (AMI) [17], [18]. To assess anterograde amnesia, they completed the Hopkins Verbal Learning test, including Immediate Recall, Delayed Recall, and Discrimination measures [19], and the Logical Memory Tests I and II from the Wechsler Memory Scale, 3rd edition [20], [21]. To assess speed of information processing, they completed the Symbol Search and the Digit-Symbol Coding subtests that make up the Processing Speed Index (PSI) of the Wechsler Adult Intelligence Scale [22], [23]. To screen executive function, they completed the Delis Kaplan Verbal Fluency Test where they were asked to generate as many words as possible in 60 sec beginning with a specific letter [24], [25].

### Statistical Analysis

Two sets of analyses were performed. First, ECT-All patients (n = 20) were compared with the ISO patients (n = 8) for HRSD-24 scores and for each of the neurocognitive performance scores at Pretreatment, Post-treatment and Follow-up using a mixed model repeated measures ANCOVA with Group (2) as a between-subjects factor and Time (3) as a within-subjects factor. For the QIDS-SR scores, which were obtained before and throughout the ECT and ISO treatment series, a Group (2)×Time (4) analysis was used, where sampling times were before the first, fourth, seventh and tenth/last treatment. When ANCOVAs indicated significant effects, between-group and within-group comparisons among means were performed, including comparisons between ECT-All vs. ISO groups and between ECT-Matched vs. ISO groups. For the Pretreatment neurocognitive measures, these means were adjusted for age and WTAR. To examine relative changes in performance after treatment between groups, Post-Treatment and Follow-up neurocognitive means were compared between-groups after adjustment for the Pretreatment score on that test as well as WTAR. Alpha level was set at p<0.05 two-tailed, even where our predicted outcomes were directional (ISO > ECT for neurocognitive measures following treatment), in an effort to limit the increase in false positive findings associated with multiple dependent variables. Cohens d estimates of effect sizes were also generated (using pooled standard deviations) for each significant group difference.

## Results

### Antidepressant effects of ECT and ISO

**Figure 1:**
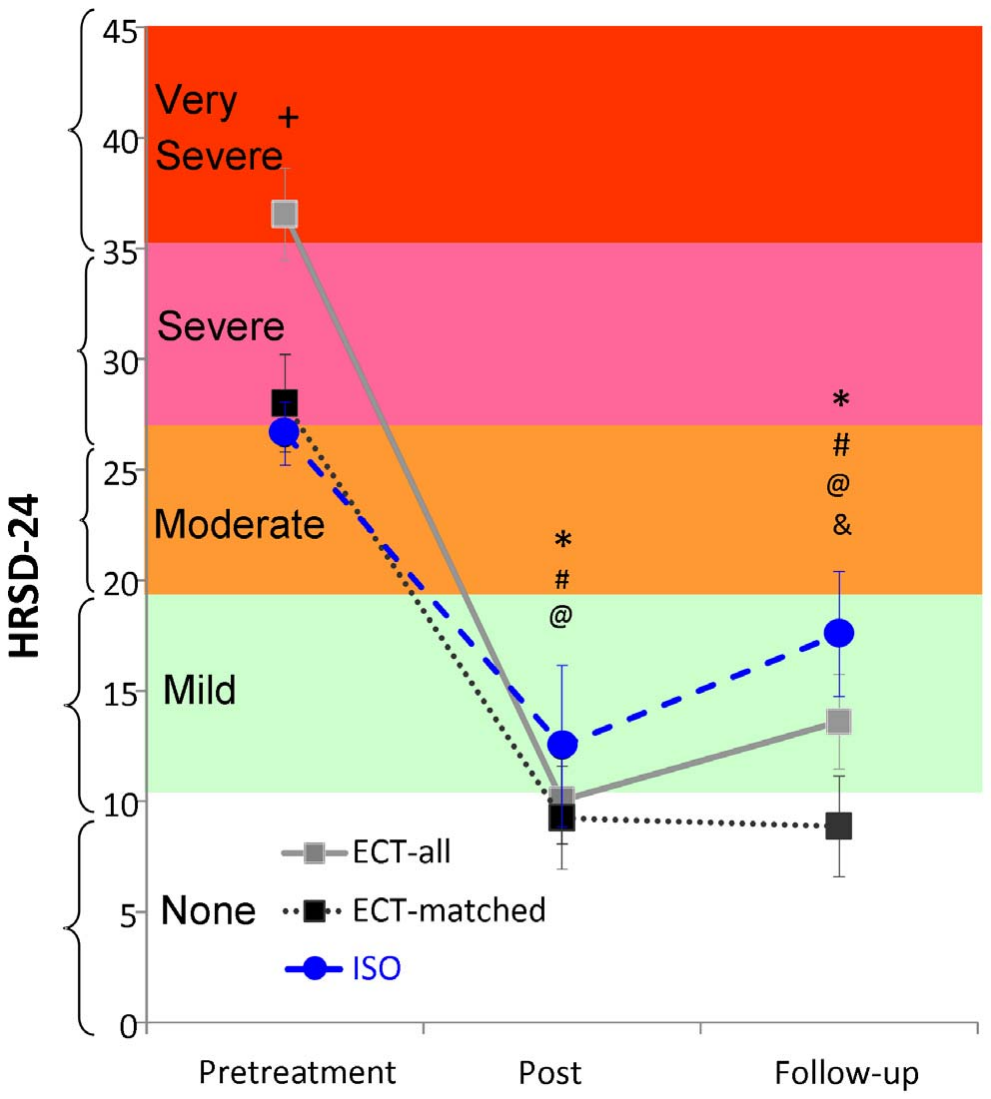
Scores on Hamilton Rating Scale for Depression (HRSD-24) at Pretreatment), 24–48 hours after the last treatment (Post-treatment) and at 4-week Follow-up. Treatment with ECT and ISO result in significant decreases in HRSD-24 depressive symptoms at both Post-treatment and Follow-up compared to Pretreatment. No significant group differences were seen at Post-treatment, but ECT-Matched maintained these low scores better than ISO at Follow-up. **+** ECT-All vs. ISO p<0.05, **&** ECT-Matched vs. ISO p<0.05, *Within-group ECT-All Change from Pretreatment p<0.001, **#** Within-group ECT-Matched Change from Pretreatment p<0.01, **@** Within-group ISO Change from Pretreatment p<0.005 at Post-treatment and p<0.05 at Follow-up.

For the HRSD-24 scores, a main effect of Times (F = 51.9, d.f. = 2,52, p<0.0001) and a significant Group X Time interaction was obtained (F = 4.58, d.f. = 1,52, p<0.05). Subsequent comparisons showed that although the ECT-All group initially had higher depression severity than the ISO group (Pretreatment mean HRSD = 36.55 vs. 26.63, p<0.008), the HRSD scores of both groups decreased significantly to similar levels at Post-treatment (Post-treatment means = 10.05 and 12.5 for ECT-All vs. ISO, group difference p = 0.57; change from Pretreatment p<0.0001 for ECT-All, and p<0.005 for ISO).These significant decreases in HRSD-24 scores persisted at 4-week Follow-up (Follow-up means = 14.52 and 17.57, group difference p = 0.30; change from Pretreatment p<0.001 for ECT-All, and p<0.033 for ISO). When ECT-All was compared with the ISO group, they did not differ significantly in HRSD-24 at Post-treatment or Follow-up, with both groups reducing mean symptom severity to the mild range (Figure 1). The ECT-Matched and ISO patients (n = 8 per group) had comparable HRSD-24 scores at Pretreatment and Post-treatment (Pretreatment means = 28.00 vs. 26.63, Post-treatment means = 9.25 vs. 12.50 for ECT Matched vs. ISO respectively, group differences p = 0.61 and p = 0.46), but the ECT-Matched subgroup maintained these low scores better than the ISO group at 4-week follow-up (Follow-up means = 8.9 vs. 17.6, respectively, p = 0.036, d = 1.39; Figure 1).

**Figure 2:**
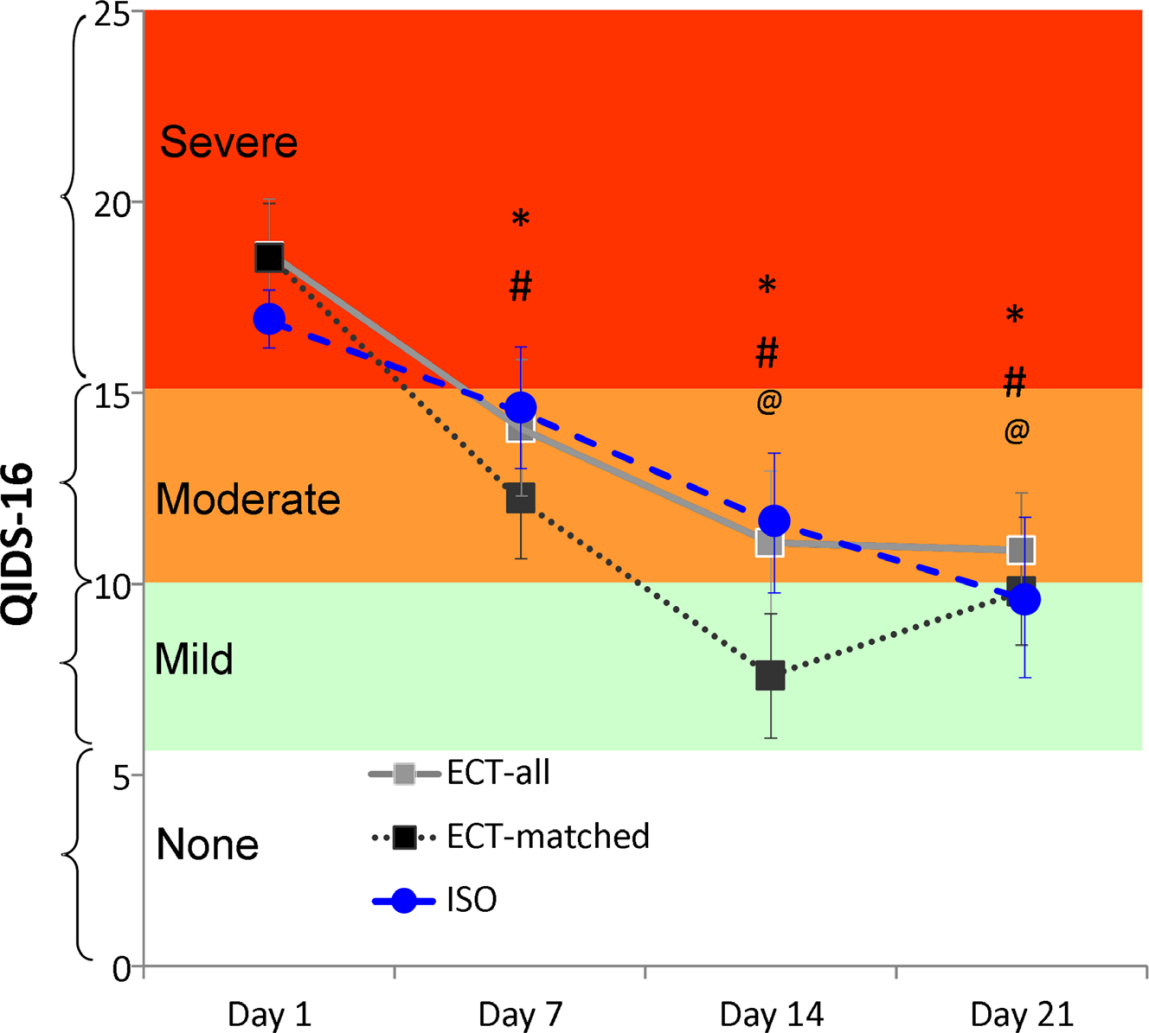
Scores on the Quick Inventory of Depression Scale (QIDS-SR16) self report form over 21 days of treatment (treatment sessions 1–10). For brevity, results are depicted showing change over each week, from Day 1 (prior to 1st treatment), Day 7 (prior to 4^th^ treatment), Day 14 (prior to 7^th^ treatment), and Day 21 (prior to 10th treatment). *Within-group ECT-All Change from Day 1 p<0.05, **#** Within-group ECT-Matched Change from Day 1 p<0.05, **@** Within-group ISO Change from Day 1 p<0.05.

Using the definition of 50% reduction in HRSD-24 depression score to identify treatment responders, 90% of ECT-All patients (18 of 20), 88% of ECT-Matched patients (7 of 8) and 75% of ISO patients (6 of 8) met this criterion at Post-treatment (Table 1). These group differences were not significant (Fishers exact test p = 0.55). Using the definition of depression remission as scores of 0–9 on the HRSD-24 (which is equivalent to scores of 0–7 on the HRSD-17 scale), 50% of each treatment group achieved remission at Post-treatment (10 of 20 ECT-All, 4 of 8 ECT-Matched and 4 of 8 ISO patients; Table 1). Likewise, self-reported depressive symptoms (QIDS-SR16) obtained prior to each of the ECT or ISO treatment sessions did not differ between the groups at any time point (Group×Time F = 0.84, d.f. = 3,70, p = 0.55), and there was similar steady improvement in all 3 groups across the treatment sessions (Main effect of Time, F = 18.65, d.f. = 3,70, p<0.0001; Figure 2).

### Neurocognitive Measures: Overall Analyses

After adjustment for differences in age and WTAR, a significant Group×Time interaction was obtained for 6 of the 8 neurocognitive measures: Hopkins Immediate Recall and Discrimination, Logical Memory I and II, Fluency and AMI (F's = 6.49, 5.23, 5.56, 7.99, 10.03 and 6.83, d.f. = 2,44, p<0.01-p<0.001). For the PSI, this interaction approached significance (p = 0.067), but the main effect of Group was more robust (F = 8.12, d.f. =  1,22, p<0.01). For Hopkins Delayed Recall, no reliable effects were seen (p>0.15). Subsequent analyses focused on comparisons of adjusted means in the 7 neurocognitive measures where overall analyses were significant (see Table 2). All means and p-values were adjusted for age and WTAR. For Post-Treatment and Follow-up scores, p-values were further adjusted for group differences in Pretreatment scores in order to examine whether groups differed significantly in any treatment-related improvements or declines in performance.

**Table 2:** Neurocognitive Means (SEM) at Pretreatment, Post-Treatment and Follow-up, Adjusted for Intellectual Level and Age.

	Pretreatment Adj. Mean (SEM)	p-value	Post-Treatment Adj. Mean (SEM)	p-value	Follow-up Adj. Mean SEM)	p-value
**Hopkins Immed Recall**						
ECT-All	45.04 (2.49)	0.41	42.02 (3.10)	***0.01***	50.83 (2.57)	**0.05**
ECT-Matched	47.63 (3.33)	0.39	51.45 (4.77)	0.40	58.23 (3.90)	0.84
ISO	49.40 (4.20)		61.08 (5.23)		60.34 (4.58)	
**Hopkins Delay Recall**						
ECT-All	44.03 (2.69)	0.18	40.65 (3.22)	0.09	50.05 (2.30)	0.65
ECT-Matched	48.25 (3.55)	0.28	52.68 (3.61)	0.78	55.17 (2.47)	0.28
ISO	51.80 (4.54)		56.76 (5.42)		55.25 (4.10)	
**Hopkins Discrim**						
ECT-All	47.55 (2.38)	0.24	35.56 (2.94)	***0.01***	46.66 (1.59)	***0.01***
ECT-Matched	50.77 (3.26)	0.48	41.42 (4.87)	0.19	49.33 (2.43)	**0.05**
ISO	53.44 (4.01)		56.05 (4.95)		55.93 (2.83)	
**Logical Memory I**						
ECT-All	9.42 (0.55)	0.23	8.69 (0.73)	***0.005***	11.45 (0.69)	**0.04**
ECT-Matched	9.00 (0.79)	0.07	10.31 (0.90)	0.21	12.91 (1.01)	0.80
ISO	10.83 (0.94)		13.49 (1.19)		15.32 (1.41)	
**Logical Memory II**						
ECT-All	10.53 (0.54)	0.85	8.18 (0.83)	***0.001***	11.59 (0.58)	***0.002***
ECT-Matched	10.54 (0.81)	0.67	10.70 (1.18)	***0.006***	13.08 (0.61)	***0.006***
ISO	10.31 (0.91)		15.40 (1.35)		15.54 (1.18)	
**Delis Kaplan Fluency**						
ECT-All	10.32 (0.63)	0.11	6.00 (0.79)	***0.001***	9.47 (0.78)	0.30
ECT-Matched	9.94 (0.98)	0.33	6.90 (1.12)	***0.001***	10.00 (0.68)	0.37
ISO	8.20 (1.06)		11.14 (1.34)		10.02 (1.39)	
**Autobiographical Mem**						
ECT-All	53.39 (1.10)	0.62	37.18 (1.88)	***0.003***	37.14 (1.93)	***0.002***
ECT-Matched	52.71 (1.94)	0.55	40.28 (2.56)	0.07	39.36 (2.41)	**0.02**
ISO	54.53 (1.85)		50.54 (3.17)		48.79 (3.45)	
**Processing Speed Index**						
ECT-All	93.22 (3.90)	**0.03**	87.40 (3.36)	**0.02**	97.89 (3.56)	**0.02**
ECT-Matched	91.16 (8.04)	0.09	91.44 (6.73)	0.23	99.71 (6.99)	0.13
ISO	112.20 (6.58)		109.75 (5.67)		119.96 (2.26)	

*All p-values are for mean comparisons versus ISO after adjustment for group differences in age and WTAR score. Post-treatment and Follow-up p-values are also adjusted for group differences in Pretreatment and thus test group differences in performance decline or improvement from Pretreatment levels.*

*Cohen's d effect sizes for significant comparisons were consistently large and ranged from 1.1 to 1.7.*

### Retrograde Amnesia: Autobiographical Memory

For age- and WTAR-adjusted AMI scores, no group differences were obtained at Pretreatment, but the ISO group had significantly better recall for dates and details of real-life autobiographical events at Post-treatment and Follow-up than both the ECT-All and the ECT-Matched groups (Table 2). Within-group comparisons indicated significant performance decrements between Pre- and Post-treatment in all groups (p<0.05 for ISO, p<0.001 for ECT-All and ECT-Matched). These decrements persisted at Follow-up. When responses after treatment were further adjusted for Pretreatment performance, the ECT-All group showed greater decline in AMI performance than the ISO group at both Post-treatment and Follow-up (p<0.003) and the ECT-Matched showed greater decline at Follow-up (p<0.02) and marginally greater decline at Post-treatment (p<0.07) compared to the ISO group.

### Executive Function

For the Verbal Fluency measure, no group differences were significant at Pretreatment or Follow-up, but the ISO group had significantly better performance immediately Post-treatment (Table 2). These Post-treatment group differences were maintained after adjustment for Pretreatment scores. Within-group comparisons indicated that the ECT-All and ECT-Matched groups showed decrements in Fluency at Post-treatment (p<0.001) while the ISO group showed a slightly improved performance (p<0.05). Both ECT groups showed significant increases in Fluency between Post-treatment and Follow-up (p<0.01) while the ISO group's Fluency score did not differ reliably between those times (p>0.19), so by Follow-up, the groups no longer differed.

### Anterograde Amnesia: Hopkins Verbal Learning and Logical Memory Tests

Among the Hopkins Verbal Learning measures, which involve memory for spoken word lists, there were no significant group differences at Pretreatment after adjustment for age and WTAR (Table 2). After further adjustment for slight differences in Pretreatment performance, the ISO group's Immediate Recall and Discrimination scores were superior to the ECT-All group at Post-treatment (p<0.01) and Follow-up (p<0.05), and their Discrimination scores at Follow-up were better than the ECT-Matched group (p<0.05). Within-group comparisons showed that both the ISO and ECT-matched groups had higher Post-treatment vs. Pretreatment scores for Immediate Recall (p<0.01 and p<0.05 respectively) while the ECT-All group did not change. For Discrimination, the ECT-All and ECT-Matched groups both declined from Pre- to Post-treatment (p<0.01 and p<0.05 respectively) while the ISO group showed no real change. As noted previously, Hopkins Delayed Recall showed no significant effects.

For the Logical Memory Tests I and II, which involve immediate and delayed recall of spoken passages, respectively, no significant group differences were obtained at Pretreatment after adjustment for age and WTAR (Table 2). At both Post-treatment and Follow-up, after adjustment for Pretreatment differences, the ISO group had better performance on both tasks than the ECT-All group (p<0.002) and better delayed recall than the ECT-Matched group (p<0.006). Within-group comparisons indicated that the ISO group had significant increases and the ECT-All group had significant decreases in Test II scores at Post-treatment vs. Pretreatment (p<0.05). Both ISO and ECT-Matched had increases in Test I scores at Post-treatment (p<0.05). All groups showed better Logical Memory I and II scores at Follow-up than at Pretreatment (p<0.05).

### Processing Speed Index

For the Processing Speed Index scores, the ISO group had significantly better performance than ECT-All at Pretreatment, Post-Treatment and Follow-up even with adjustment for WTAR and age (p<0.03; Table 2) but were only marginally different from ECT-Matched (p<0.09). Only the ECT-All group showed a significant drop in PSI score at Post-treatment (p<0.05), and this led to a significant group difference between the ISO and ECT-All group at Post-treatment after further adjustment for Pretreatment scores. All groups showed higher PSI scores at Follow-up vs. Post-treatment (p<0.05).

## Discussion

### Antidepressant Efficacy

The results of this open label study supported our primary hypothesis that a series of 10 sessions of deep anesthesia with ISO is effective as an antidepressant intervention for patients with medication-refractory depression. The improvement in HRSD-24 scores (including percent of patients achieving remission) and QIDS-SR16 scores immediately after completion of treatment was similar in the nonstandard ISO treatment compared to the standard ECT treatment. Although there was somewhat better maintenance of the decrease in HRSD-24 depressive symptom scores among the 8 severity-matched ECT patients one month later, the ISO group still showed significant improvement even at that later time point.

The antidepressant effect of ISO indicated by these observations is very similar to the original reports by Langer et al [9], [10]. The explanation for why our results were more positive than other later studies may be due to closer adherence to the original protocols, particularly in patient age, dose/duration of each ISO session, and use of more ISO treatment sessions. As suggested by the QIDS-SR16 self-report data in Figure 2, much of the beneficial effect of ISO occurred in sessions 5–10, while Garcia-Toro et al terminated their treatments after only 4 sessions, and they used sevoflurane which might be less effective than ISO, especially if sustained EEG burst suppression is needed for a clinical benefit [14]. We also restricted our sample to persons aged 65 and younger, in part because of the increased likelihood of adverse blood pressure and cardiovascular concerns in elderly patients, like those studied by Greenberg et al [13]. We also were able to reduce side effects (nausea, vomiting, and disorientation) and shorten the time to full recovery after each session by use of the ANEclear**™**.

### Cognitive Effects

The neurocognitive assessments made as part of this study indicate that even with 10 sessions of ISO anesthesia over a 3 week period, there was no significant performance decrement on any of the traditional neurocognitive measures. In fact, the ISO patients showed significant improvements in word fluency, nonverbal processing speed, logical memory, and immediate and delayed recall of newly learned verbal material after the intervention. These improvements are probably a result of the combined effects of decreased depressive state (which has been linked to response slowing and other performance deficits) and practice [6], [21], [26]. The one measure where there was any suggestion of a performance decline with ISO treatment was the Autobiographical Memory Interview. This scale has intrinsic flaws because the responses given at the initial interview are designated as correct without verification. Consequently, when retesting occurs, the individual's performance can worsen but cannot show improvement, and any answers that differ from the original ones (even if the patient has remembered more accurately when retested) are scored as errors. Recently, Semkovska and colleagues found that when retested after 2 months, healthy controls as well as depressed patients never treated with ECT showed similar mild declines in AMI performance, which they labeled as consistency errors rather than true memory deficits [18]. For the ISO patients, their small post-treatment decrements in AMI scores were similar in magnitude to expected test-retest consistency errors, and their performance was superior to the ECT patients both immediately after the last treatment and one month later. Thus, multiple sessions of ECT led to persistent AMI deficits that were not seen after multiple sessions of ISO.

The effectiveness of bifrontal ECT on depressive symptoms was never in doubt [2], [3], [4], [5], but our results on the time course and relative magnitude of other neurocognitive decrements are important to note. When tested within 24–48 hours after their last ECT session, the ECT-All group also showed declines in word fluency, immediate and delayed recall of verbal material, ability to discriminate between words previously heard versus other words, and processing speed. The ECT-Matched group had fewer performance deficits, but still showed significant reductions in word fluency and discrimination at Post-Treatment, and they failed to match the practice-related improvement seen in the ISO group for delayed recall of passages. After one month, however, performance of the ECT patients on all tests except for the AMI returned to or exceeded Pretreatment levels. This is consistent with the meta-analysis by Semkovska and McLoughlin [6] , which noted that neurocognitive deficits linked to bifrontal ECT were mainly limited to the first 3 days post-treatment, and at 15 days post-treatment, there was evidence of improvement in both memory and executive function [6]. Comparisons between means adjusted for pretreatment task performance and intellectual level indicated, however, that the improvements 4 weeks after treatment in the ISO group were greater than the full ECT sample for several cognitive measures, including immediate recall of newly learned words and passages, word discrimination, delayed recall of passages, and processing speed. Possibly the ISO patients were able to benefit more from practice at the testing done 24–48 hours after the last treatment when many ECT patients were experiencing significant cognitive impairment. Also, the ECT-Matched group had improvements as great as the ISO group for all measures except word discrimination, delayed recall of passages and autobiographical memory. This suggests that initial depression severity contributes to lesser post-treatment improvement in immediate recall of words and passages and processing speed. Nevertheless, we cannot rule out a residual effect of ECT influencing performance on some cognitive measures in addition to AMI at 4-week follow-up.

### Study Limitations

There are several study limitations to acknowledge. The study was open-label rather than randomized, and the group sizes were modest. Replication in a larger clinical trial is needed to confirm these findings. More of the severely depressed patients chose ECT than ISO, which was managed by creating a smaller ECT-Matched subgroup to examine along with the full ECT and ISO groups. The effectiveness of ISO for the most severely depressed patients is still uncertain. Also, some of the ECT patients had 8 or 12 rather than 10 treatments, titrated to clinical effectiveness, but our IRB approval restricted the ISO treatment series to 10. This factor may have contributed to the slightly better antidepressant outcome for the ECT-Matched patients at 4-week follow-up. Finally, all of the patients in the study had medication-refractory moderate to severe depression, so that future research is needed to examine whether ISO anesthesia may be an effective treatment in milder and medication-responsive depression. This small open-label study indicates the ISO may be another option for patients with moderate to severe depression (both with MDD and BPD diagnoses) who find medications ineffective or are unable to tolerate their side effects. Like transcranial magnetic and direct current stimulation, magnetic seizure therapy and other novel treatment options, it is important to explore the comparable efficacy as well as side effect profiles of novel alternatives to ECT for seriously depressed patients [27], [28], [29]. It would also add value if future studies examine whether these alternative therapies, including ISO anesthesia, convey their antidepressant effects through the same or different pathways as ECT, such as by decreasing frontal cortical connectivity [30] or by altering the transcription and expression of the same neuronal and immune genes [31].

### Implications

This small study counters intervening negative studies and confirms the original research showing that a series of 10 treatment sessions with deep inhalation isoflurane anesthesia has an antidepressant effect in moderate to severe medication-refractory depression approaching that of bifrontal ECT. Neurocognitive performance of ISO-treated patients was superior to ECT-treated patients immediately after the last treatment (when only ECT was associated with worsened performance relative to pretreatment). ISO patients continued to perform better 4 weeks later, although performance in ECT patients showed considerable improvement between those tests. With this additional supportive evidence, a larger clinical trial is strongly indicated, preferably one designed to examine whether ECT and ISO have their effects through shared physiological pathways.
