# Correction: Antidepressant and Neurocognitive Effects of Isoflurane Anesthesia versus Electroconvulsive Therapy in Refractory Depression

**DOI:** 10.1371/journal.pone.0175668

**Published:** 2017-04-06

**Authors:** Howard R. Weeks, Scott C. Tadler, Kelly W. Smith, Eli Iacob, Mikala Saccoman, Andrea T. White, Joshua D. Landvatter, Gordon J. Chelune, Yana Suchy, Elaine Clark, Michael K. Cahalan, Lowry Bushnell, Derek Sakata, Alan R. Light, Kathleen C. Light

The following information is missing from the Competing Interests section: At the time of the study, author DS had an equity interest in Anecare, a commercial company, which holds the license to the ANEclear device that was used in the study. There are no patents, products in development or marketed products to declare. This does not alter our adherence to all the PLOS ONE policies on sharing data and materials.
